# Increased cognitive load in immersive virtual reality during visuomotor adaptation is associated with decreased long-term retention and context transfer

**DOI:** 10.1186/s12984-022-01084-6

**Published:** 2022-10-05

**Authors:** Julia M. Juliano, Nicolas Schweighofer, Sook-Lei Liew

**Affiliations:** 1 Neuroscience Graduate Program, University of Southern California, 2250 Alcazar St., CSC 133, Los Angeles, CA 90089 USA; 2 Biokinesiology and Physical Therapy, University of Southern California, Los Angeles, CA USA; 3 Chan Division of Occupational Science and Occupational Therapy, University of Southern California, Los Angeles, CA USA; 4 USC Stevens Neuroimaging and Informatics Institute, Department of Neurology, Neurology, University of Southern California, Los Angeles, CA USA

**Keywords:** Immersive virtual reality, Head-mounted display, Cognitive load, Visuomotor adaptation, Long-term retention, Context transfer

## Abstract

**Background:**

Complex motor tasks in immersive virtual reality using a head-mounted display (HMD-VR) have been shown to increase cognitive load and decrease motor performance compared to conventional computer screens (CS). Separately, visuomotor adaptation in HMD-VR has been shown to recruit more explicit, cognitive strategies, resulting in decreased implicit mechanisms thought to contribute to motor memory formation. However, it is unclear whether visuomotor adaptation in HMD-VR increases cognitive load and whether cognitive load is related to explicit mechanisms and long-term motor memory formation.

**Methods:**

We randomized 36 healthy participants into three equal groups. All groups completed an established visuomotor adaptation task measuring explicit and implicit mechanisms, combined with a dual-task probe measuring cognitive load. Then, all groups returned after 24-h to measure retention of the overall adaptation. One group completed both training and retention tasks in CS (measuring long-term retention in a CS environment), one group completed both training and retention tasks in HMD-VR (measuring long-term retention in an HMD-VR environment), and one group completed the training task in HMD-VR and the retention task in CS (measuring context transfer from an HMD-VR environment). A Generalized Linear Mixed-Effect Model (GLMM) was used to compare cognitive load between CS and HMD-VR during visuomotor adaptation, t-tests were used to compare overall adaptation and explicit and implicit mechanisms between CS and HMD-VR training environments, and ANOVAs were used to compare group differences in long-term retention and context transfer.

**Results:**

Cognitive load was found to be greater in HMD-VR than in CS. This increased cognitive load was related to decreased use of explicit, cognitive mechanisms early in adaptation. Moreover, increased cognitive load was also related to decreased long-term motor memory formation. Finally, training in HMD-VR resulted in decreased long-term retention and context transfer.

**Conclusions:**

Our findings show that cognitive load increases in HMD-VR and relates to explicit learning and long-term motor memory formation during motor learning. Future studies should examine what factors cause increased cognitive load in HMD-VR motor learning and whether this impacts HMD-VR training and long-term retention in clinical populations.

**Supplementary Information:**

The online version contains supplementary material available at 10.1186/s12984-022-01084-6.

## Background

Immersive virtual reality using a head-mounted display (HMD-VR) has been increasingly used for motor learning and rehabilitation purposes [1]. Recent technological developments in HMD-VR have made these devices obtainable at relatively low costs. Driving factors for using HMD-VR in motor rehabilitation include the ability to replicate and even go beyond the real world, allowing for researchers and clinicians to have increased control of the training environment and tailor it for each patient’s needs [1]. Moreover, use of these devices have been shown to increase motivation and engagement and allow for patients to spend more time actively engaged in therapy [2–4]. However, while some applications of interventions performed in HMD-VR have shown to be either comparable or superior to the same intervention performed in a conventional rehabilitation setting [5–7], other applications have been less effective than conventional environments [8–10]. There is also inconclusive and conflicting evidence about whether motor skills learned in HMD-VR will transfer to the real world, and reasons underlying a lack of contextual transfer are unclear [11, 12]. These findings suggest that there may be instances when the use of HMD-VR could potentially result in less effective motor learning. Understanding what makes learning motor skills in HMD-VR different from learning motor skills in the real world can better inform the design of HMD-VR applications so that they can be transferred to new environments and be more effective for clinical populations.

Evidence suggests that motor skills are learned differently between HMD-VR and conventional environments [11]. A first potential difference is the movement kinematics within each environment. Studies comparing movements made by individuals both with and without motor impairments found movements in an HMD-VR environment to be slower and less smooth compared to a real-world environment [13, 14]. These results suggest that movement parameters, especially when a virtual avatar is present, should be monitored in HMD-VR motor learning applications. A second potential difference is the underlying motor learning mechanisms used in each environment. HMD-VR has been shown to recruit greater explicit, cognitive strategies during visuomotor adaptation compared to a conventional computer screen (CS), suggesting that the process by which motor skills are acquired in HMD-VR may be different than in conventional environments [15]. Specifically, these findings suggest that motor learning in HMD-VR may require additional cognitive processing compared to conventional environments.

Converging evidence supports this and shows that cognitive load increases during highly stressful and complex motor tasks in HMD-VR compared to CS environments [16–18]. Cognitive load is the amount of information that can be held in working memory at one time. The theoretical construct of cognitive load suggests that novel information (e.g., a new visuomotor mapping) can be encoded in long-term memory when the load on working memory is within working memory limits [19]. Increased cognitive load in HMD-VR, measured by the attentional demands of a secondary task during complex motor tasks, is shown to decrease motor performance compared to CS [16]. However, it is unclear whether HMD-VR-related increases in cognitive load and decreases in motor performance result in decreased long-term motor memory formation, which is critical for clinical uses. Based on the theoretical framework of cognitive load, we hypothesized that an increase in cognitive load during HMD-VR motor tasks would be related to a decrease in motor memory formation, resulting in both decreased long-term retention and context transfer.

To examine this hypothesis, we first aimed to examine differences in cognitive load between HMD-VR and CS during a visuomotor adaptation task. Visuomotor adaptation is thought to be an error-driven process driven by competing explicit and implicit mechanisms updated on a trial-by-trial basis [20, 21]. Explicit mechanisms are thought to be important early in adaptation and reflect the cognitive strategies used to adapt to experienced perturbations. Implicit mechanisms, on the other hand, develop over the course of adaptation and reflect a recalibration of an internal model, thought to represent a mapping between the desired goal and the appropriate motor response to accomplish the goal [22, 23]. Explicit processes are also found to be affected by a secondary cognitive task, while implicit processes are not affected by the interference [24]. As previously mentioned, HMD-VR has been shown to recruit greater explicit, cognitive strategies during visuomotor adaptation. However, it is not known whether the recruitment of greater cognitive strategies is related to increased cognitive load. Thus, the second aim of this study is to examine the relationship between explicit mechanisms and cognitive load during visuomotor adaptation. This was done by combining an established visuomotor adaptation task with a dual-task probe measuring attentional demands to assess cognitive load and by training individuals in either an HMD-VR or CS environment. After training on the visuomotor adaptation task with the dual-task probe, participants returned after a 24-h retention period to measure long-term retention as well as HMD-VR context transfer. To address the third aim of this study, we then examined the relationship between cognitive load and long-term motor memory and context transfer of the overall adaptation. By measuring attentional demands throughout the visuomotor adaptation process, we then examined whether the cognitive load during training is related to long-term retention and context transfer.

## Methods

### Participants

Forty-one participants were recruited for the study. Two participants did not complete the experiment due to an inability to follow instructions (CS: N = 2). From the thirty-nine participants who completed the experiment, one was removed due to a previous knee injury that hindered their ability to press the foot pedal on the dual-task probe (CS: N = 1) and two were removed due to missing greater than 25% of the dual-task probe trials (HMD-VR: N = 2). This resulted in thirty-six participants included in the final training analysis, with N = 12 training in the CS environment and N = 24 training in the HMD-VR environment (22 female/14 male; aged: M = 26.3, SD = 4.6). For the final retention analysis, participants training in the HMD-VR environment were equally divided into two groups, where half of participants continued in the HMD-VR environment (N = 12), and the other half transferred to the CS environment (N = 12; see *Retention* for details). Eligibility criteria included right-handed individuals with no neurological impairments and normal or corrected-to-normal vision. Data were collected in-person during the COVID-19 pandemic, and all participants wore surgical masks for the duration of the experiment. Written informed consent was electronically obtained from all participants to minimize in-person exposure time at the lab. The experimental protocol was approved by the USC Health Sciences Campus Institutional Review Board and performed in accordance with the 1964 Declaration of Helsinki.

### Experimental apparatus

**Fig. 1:**
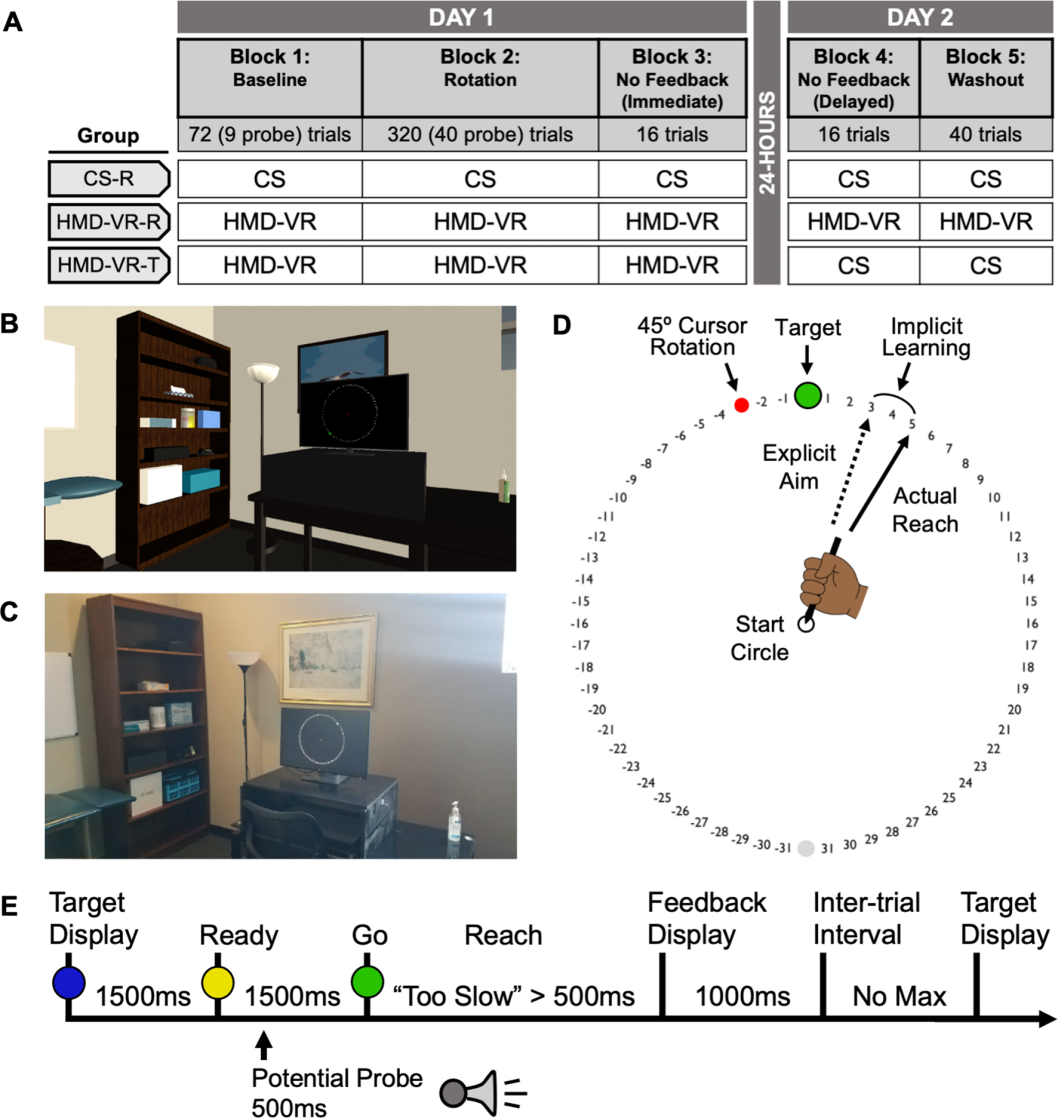
Experimental paradigm. **A** Experimental design. Participants trained on a visuomotor adaptation task in either **B** an HMD-VR environment or **C** a CS environment. **D** Visuomotor adaptation task with a 45° counterclockwise rotation **E** combined with dual-task probe. After finding the start circle, a blue target with numbers flanking the target would appear, and participants were asked to report where they planned to aim. Participants remained at the start circle for 3000 ms as the target changed from blue to yellow to green, and then made quick reaching movements through the target. Once crossing the invisible outer circle, the endpoint location of the reach (or rotated reach) would be displayed as a red cursor. On some of the trials, after the target turned yellow, an auditory cue was played 500 ms after the target turned yellow and participants responded by quickly pressing a foot pedal under their right foot. The reaction time of the foot pedal press was used as the measure of cognitive load

Participants completed an established visuomotor adaptation task modified with a dual-task probe to measure attentional demands (Fig. 1A) [15, 25, 26]. The task was completed in either a CS or HMD-VR environment. The HMD-VR environment used an Oculus Quest (Fig. 1B) and showed an environment modeled after the CS environment (Fig. 1C). In both environments, participants grasped a digitalized stylus with their right hand and reached for one of eight pseudorandomized targets using a tablet (Wacom Intuos4 Extra Large). Movement trajectories were sampled at 60 Hz in both CS and HMD-VR environments. To control for potential differences in movement kinematics, participants were unable to see their bodies in either environment (i.e., bodies were covered using a cloth cover in CS and no virtual avatar was provided in HMD-VR). Instead, visual feedback of the stylus was provided in the form of a red circular cursor (5 mm diameter) and displayed on an upright computer monitor set on a large box which was placed over the tablet. The computer monitor located in the CS environment was a 24.1 inch, 1920 × 1200 pixel resolution computer monitor (HP) located 23 cm above the tablet. The HMD-VR environment replicated the dimensions of the computer monitor as well as all the other aspects of the room and was designed using the game engine development tool, Unity 3D (version 2019.4.11f1). There were no differences between CS and HMD-VR environments in how participants were able to move. Participants were given the opportunity to explore the virtual environment before beginning the task.

### Experimental design

The experiment took place over two days (Fig. 1A). On the first day, participants completed two long training blocks followed by a short retention block (Blocks 1–3). On the second day, participants completed a 24-h retention block (Block 4) followed by an adaptation wash-out block (Block 5). Participants completed 8 familiarization trials before starting Block 1*.*

*Training* On the first day, participants completed familiarization and training (8 trials followed by Blocks 1–2, which spanned a total of 392 trials). At the start of each trial, participants moved to the start circle (7 mm diameter) located at the center of the monitor using a guiding circle that got smaller as they got closer to the center. After remaining at the start circle for 1000 ms (ms), the cursor appeared along with one of eight pseudorandom targets (10 mm in diameter) flanked by positive and negative numbers spaced 5.625° apart (Fig. 1D). The targets were located on an invisible outer circle with a diameter of 28 cm and spaced 45° apart (0°, 45°, 90°, 135°, 180°, 225°, 270°, 315°). During each trial, the target turned three different colors. The target initially appeared blue, signaling participants to verbally report to the experimenter where they planned to aim. After 1500 ms, the target then turned yellow, signaling participants to “get ready”. Lastly, after another 1500 ms, the target turned green, signaling participants to reach for the target. Participants were instructed to make “fast, slicing movements” towards the target and to shoot through the target. Reaction time (RT) was defined as the time between when the target turned green and when the cursor left the start circle, and movement time (MT) was defined as the time between when the cursor left the start circle and when the cursor crossed the invisible outer circle. If the MT exceeded more than 500 ms, participants were given an auditory warning that said, “Too Slow”. After crossing the invisible outer circle, participants received auditory feedback based on the accuracy of their reach (i.e., a pleasant “ding” if the cursor crossed the target or an unpleasant “buzz” if the cursor did not cross the target). Participants were also given visual endpoint feedback at the location where the cursor crossed the invisible outer circle for 1000 ms before starting the next trial (Fig. 1E).

Pseudorandomly, once per cycle (8 trials/cycle), an auditory cue (i.e., a horn) would sound 500 ms after the target turned yellow. Participants were instructed to press a foot pedal located under their right foot as quickly as possible, with the goal being to still reach for the target with their stylus as soon as it turned green. Importantly, participants were also instructed to prioritize reaching for the target over pressing the foot pedal. This was adapted from Goh et al., 2014, which validated this dual-task probe measuring attentional demands during a discrete motor task [26]. Given that both the motor plan and the response to the probe are held in working memory during this time, the RT to the secondary dual-task probe task was used as a measure of the cognitive load dedicated to the primary reaching task.

Before beginning training, participants watched an instructional video describing the experimental design and providing an example of a trial. Participants were instructed to begin by aiming directly at the target but told that at some point in the task, they may need to aim somewhere other than the target to make the cursor land on the target. After completing a familiarization cycle (8 trials) where they were given clarifying instructions if needed, participants then began training with the Baseline block (Block 1: 72 trials) where they made unperturbed reaches to targets. Without additional instruction, the Rotation block began where a 45° counterclockwise perturbation was introduced. Participants needed to counteract the perturbation for the cursor to land on the target (Block 2: 320 trials). Participants were given a two-minute break every 100 trials. During these breaks, participants in the HMD-VR group were allowed to remove the HMD-VR headset if desired.

*Retention* After completing training, participants completed a No Feedback (Immediate) block split into Strategy and No-Strategy cycles, counterbalanced across participants (Block 3: 16 trials). In the Strategy cycle, participants were instructed to continue using whatever strategy they developed at the end of training to get the cursor to land on the target. In the No-Strategy cycle, participants were told to refrain from using any aiming strategy and instead aim directly to the target. In both cycles, numbers flanking the target still appeared; however, participants were told to no longer report their aim and that no feedback would be provided. The average movement angle in the Strategy cycle was used to determine how much of the adaptation was retained. The average movement angle in the No-Strategy cycle was used to examine the implicit processes contributing to retention. The difference between the average movement angles in the Strategy and No-Strategy cycles was calculated to examine the explicit processes contributing to retention. Use of the Strategy and No-Strategy cycles, called process dissociation procedure, assumes that participants can disengage from using a cognitive strategy when instructed to do so [27, 28]. This concluded the first day of the experiment. Participants returned the next day, after a 24-h retention interval period, and completed No Feedback (Delayed) (Block 4: 16 trials) and Washout (Block 5: 40 trials) blocks. The No Feedback (Delayed) block was identical to the No Feedback (Immediate) block with Strategy and No Strategy cycles, and the Washout block was identical to the Baseline block, with the exception that participants no longer had to report their aim.

Participants who trained in the CS environment completed both No Feedback blocks as well as the Washout block (Blocks 3–5) in the CS environment to measure long-term retention (CS-R). Half of the participants who trained in the HMD-VR environment completed the two No Feedback and Washout blocks in the HMD-VR environment to measure long-term retention of HMD-VR (HMD-VR-R), while the other half completed these blocks in the CS environment to measure context transfer from HMD-VR to CS (HMD-VR-T). As noted above, participants were randomly assigned to groups.

### Movement analysis

All kinematic data was recorded by Unity 3D for both CS and HMD-VR environments. To assess overall adaptation, we used the endpoint hand angle, which was measured as the moment when the cursor crossed the invisible outer circle. Targets were rotated to a common reference angle set at 0°, and endpoint hand angle was calculated as the difference between the reference angle and the line between the origin and the endpoint of the hand. To assess explicit adaptation, we used aiming angle, which was measured as the reported aim multiplied by 5.625° (i.e., the degrees separating each number on the invisible outer circle). Positive angles indicate a clockwise direction from the target and negative angles indicate a counterclockwise direction from the target. To assess implicit adaptation, we calculated the difference between aiming angle and hand angle. Changes in hand angle, aiming angle, and implicit adaptation were calculated as individual means across 8 trials per cycle. All data are reported in endpoint hand angles, not target errors.

### Statistical analysis

Statistical analyses were conducted using R (version 3.6.3). Trials were excluded if participants failed to report the aiming angle (0.45% of trials), moved before the target turned green (2.92% of trials), or movements were made in the wrong direction (i.e., > 120° from target or rotation angle; 0.69% of trials) [29]. We also removed trials where the reaction time or movement time was greater than 3 standard deviations from the participants mean (2.87% of trials) [30, 31]. The two HMD-VR groups were combined for the training analysis but separated for the retention analysis.

To compare attentional demands between CS and HMD-VR during visuomotor adaptation, we used a Generalized Linear Mixed-Effect Model (GLMM) with individual participants as a random-effect variable. The RT (ms) on the dual-task probe (i.e., cognitive load) was used as the response variable, while Training Environment, Cycle, and a Training Environment × Cycle interaction term were used as fixed-effect variables. We used the function “glmer” with an Inverse Gaussian family in the lme4 R package [32]. The significance of each parameter was assessed by the Wald z-statistic.

To quantify training, we used unpaired t-tests and compared the mean hand angle, aiming angle, implicit adaptation, RT, and MT between CS and HMD-VR environments during Baseline and Rotation blocks. We also examined differences between training environments at the End of Baseline, defined as the last cycle of the Baseline block, as well in Early and Late Adaptation, defined as the mean of the first and last four cycles of the Rotation block, respectively.

To quantify retention and context transfer, we examined both immediate and delayed forgetting, calculated by subtracting Late Adaptation from the No Feedback (Immediate) and No Feedback (Delayed) blocks, respectively [33]. One-factor ANOVAs were used to compare group differences and individuals with movement angle greater than two standard deviations from the group mean in either the Strategy or No-Strategy cycles of the No Feedback blocks were excluded from this part of the analysis. Additionally, given that our a priori hypothesis was that cognitive load would affect long-term retention and context transfer, we ran post hoc analyses between groups using unpaired t-tests for delayed forgetting. All measures are reported as mean $\pm$ standard deviations in Additional file 1: Table S1 and S2 and significance was considered at p < 0.05.

## Results

### Cognitive load is greater across visuomotor adaptation in HMD-VR compared to CS

**Fig. 2:**
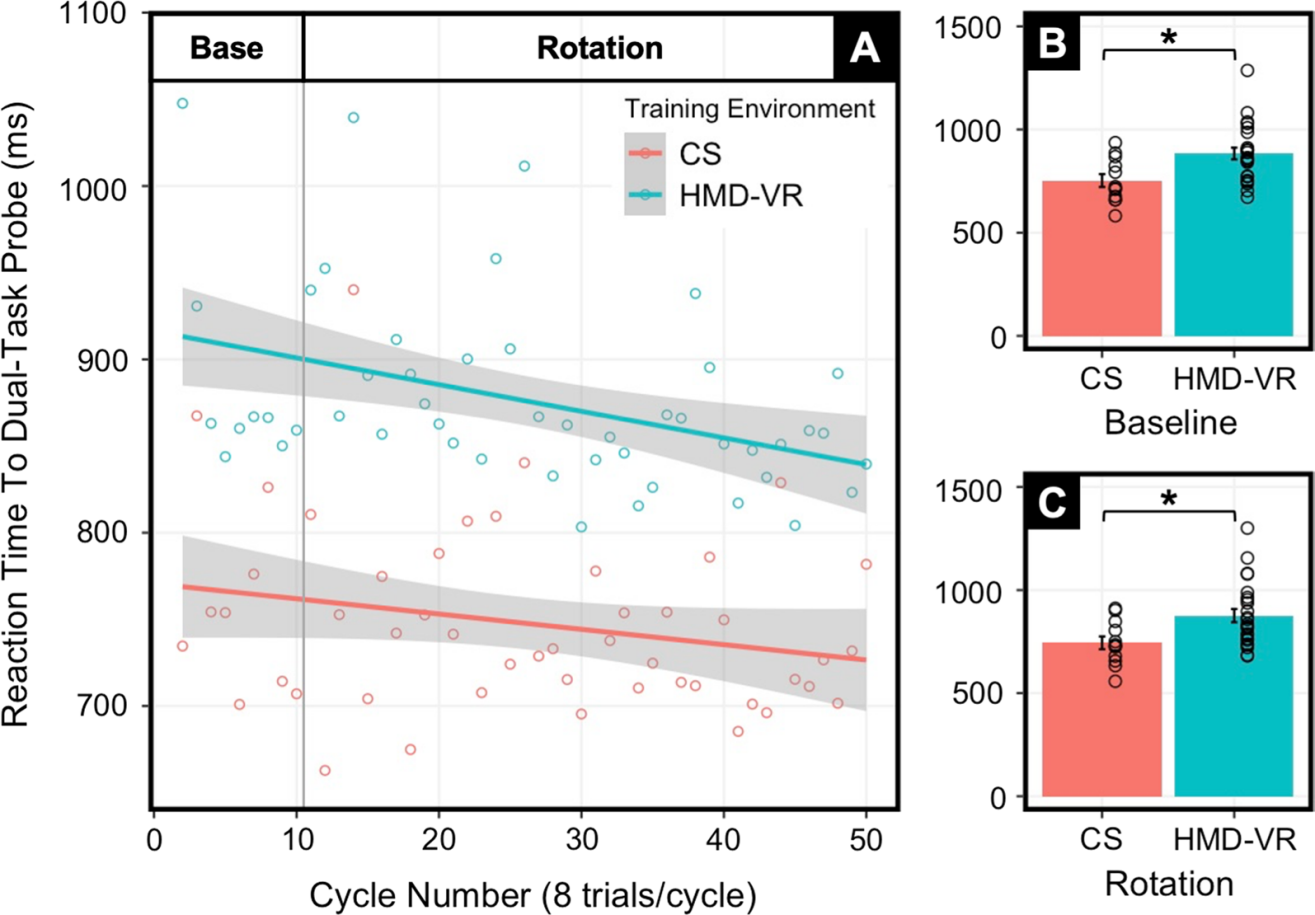
Results between HMD-VR and CS training environments on dual-task probe. **A** Cognitive load measured as the reaction time to the dual-task probe across the visuomotor adaptation task for participants training in either CS or HMD-VR environments. GLMM indicated both Cycle [${\widehat{{\varvec{\upbeta}}}}_{1}$ = − 0.83, p = 0.009] and Training Environment [${\widehat{{\varvec{\upbeta}}}}_{2}$ = 167.39, p < 0.0001] were significantly related to cognitive load where training in HMD-VR was related to increased cognitive load. Dots represent the average cognitive load across individuals for each cycle. Furthermore, average cognitive load was greater for the HMD-VR environment during **B** the Baseline block [t(27.5) = − 3.12, p = 0.004] and **C** the Rotation block [t(30.4) = − 2.96, p = 0.006] compared to the CS environment; dots represent individual averages across blocks and error bars indicate standard error. p < 0.05^*^

Cognitive load was larger in HMD-VR than in CS, as shown by the significant coefficient of Training Environment [${\widehat{{\varvec{\upbeta}}}}_{2}$ = 167 ± 38, p < 0.0001]. In addition, cognitive load decreased over the course of training in both environments as shown by the negative coefficient of Cycle [${\widehat{{\varvec{\upbeta}}}}_{1}$ = − 0.83 ± 0.32, p = 0.009] (Fig. 2A; Additional file 1: Table S3). Cognitive load was also greater in HMD-VR than in CS during the Baseline block [Fig. 2B; t(27.5) = − 3.12, p = 0.004] and during the Rotation block [Fig. 2C; t(30.4) = − 2.96, p = 0.006]. These results suggest that the attentional demands of participants in HMD-VR were greater, both with and without perturbed reaches, compared to CS visuomotor adaptation.

### Overall visuomotor adaptation between HMD-VR and CS environments

**Fig. 3:**
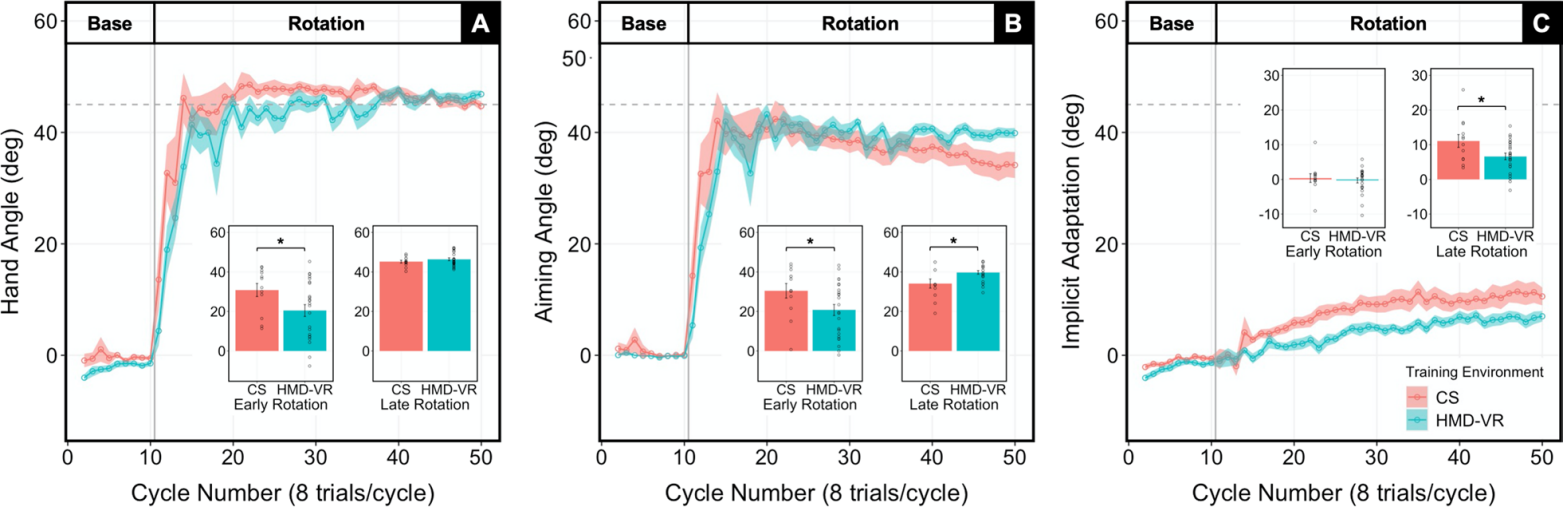
Results between HMD-VR and CS training environments on visuomotor adaptation task. Means (M) and standard errors (SE) are plotted across cycles. Insert bar graphs show group M and SE as well as individuals means during early (first 4 cycles) and late (last 4 cycles) adaptation. **A **Hand angle, measured as the angle at which the hand crossed the outer circle, for the HMD-VR and CS training environments. The hand angle was significantly larger for CS compared to HMD-VR early in adaptation [t(27.8) = 2.31, p = 0.028]; however, by late adaptation there were no differences between groups [t(29.6) = -1.19, p = 0.244]. **B **Aiming angle, measured as the aiming number reported by the participant, for the HMD-VR and CS training environments. The aiming angle was significantly larger for CS early in adaptation [t(23.7) = 2.09, p = 0.047] but was significantly larger for HMD-VR late in adaptation [t(14.5) = -2.28, p = 0.038]. **C** Implicit adaptation, measured as the difference between aiming angle and hand angle, for the HMD-VR and CS training environments. While there was no significant difference in implicit adaptation early in adaptation [t(18.7) = 0.47, p = 0.642], there were significant group differences late in adaptation [t(17.0) = 2.14, p = 0.047], where implicit adaptation in CS was larger than HMD-VR. p < 0.05^*^

To examine overall visuomotor adaptation between training environments, we compared hand angle between HMD-VR and CS across the entire Baseline block and the entire Rotation block (Fig. 3A).

*Baseline* Performance was similar between environments before the perturbation was introduced, with no difference in hand angle at the End of Baseline [t(34.0) = 1.55, p = 0.130]. Additionally, there were no significant differences between training environments at the End of Baseline for RT [t(33.8) = − 1.19, p = 0.241] and MT [t(18.8) = − 0.91, p = 0.376].

*Rotation* There was a significant difference in hand angle across the entire Rotation block, where overall visuomotor adaptation was greater for CS than in HMD-VR [t(32.8) = 2.15, p = 0.039]. In Early Rotation, the hand angle was larger in CS than in HMD-VR [t(27.8) = 2.31, p = 0.028]. However, there was no difference in hand angle in Late Rotation [t(29.6) = − 1.19, p = 0.244]. There were no significant differences between training environments in RT [t(29.9) = − 1.09, p = 0.286] and MT [t(21.5) = − 0.67, p = 0.512] across the entire Rotation block. Lastly, there were no significant differences between training environments for RT [t(32.2) = − 1.07, p = 0.293) and MT [t(29.0) = − 1.67, p = 0.105] in Early Rotation, nor in Late Rotation [RT: t(33.9) = − 1.14, p = 0.263; MT: t(19.2) = − 0.34, p = 0.739].

### Explicit and implicit contributions to visuomotor adaptation between HMD-VR and CS environments

Before examining the relationship between explicit mechanisms and cognitive load, we examined the relative contributions of explicit and implicit mechanisms across the visuomotor adaptation task between training environments. Aiming angle was used to measure the contributions of explicit, cognitive mechanisms, and implicit adaptation was used to measure the contributions of implicit mechanisms.

*Baseline* There were no differences in aiming angle between environments at the End of Baseline [t(18.8) = 0.48, p = 0.640]. There was also no difference in implicit adaptation between environments at the End of Baseline [t(30.0) = 1.11, p = 0.277]. These results suggest that explicit and implicit mechanisms were similar between environments before the perturbation was introduced.

*Rotation* We examined aiming angle across the entire Rotation block and found no difference between training environments [t(18.1) = − 0.27, p = 0.787]. However, in Early Rotation, aiming angle was larger in CS than in HMD-VR [t(23.7) = 2.09, p = 0.047]. Conversely, in Late Rotation, aiming angle was larger in HMD-VR than in CS [t(14.5) = − 2.28, p = 0.038]. These results show that while no differences are seen across the entire adaptation block in aiming angle, this is the result of aiming angle being greater early in adaptation for the CS environment but greater later in adaptation for the HMD-VR environment (Fig. 3B).

Throughout the Rotation block, implicit adaptation was larger in CS than in HMD-VR [t(17.1) = 2.57, p = 0.020]. In Early Rotation, there was no significant difference between environments [t(18.7) = 0.47, p = 0.642]. In Late Rotation, implicit adaptation was significantly larger in CS than in HMD-VR [t(17.0) = 2.14, p = 0.047]. These results suggest that the differences in implicit adaptation between training environments are driven by differences developed later in adaptation (Fig. 3C).

### Relationship between cognitive load and explicit mechanisms during visuomotor adaptation

**Fig. 4:**
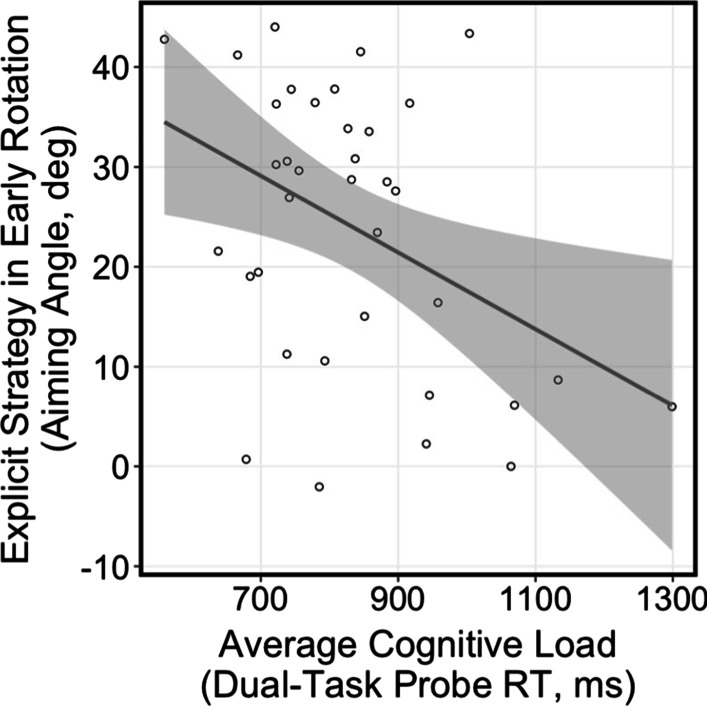
Average cognitive load plotted against aiming angle during Early Rotation. Increased cognitive load related to decreased cognitive, explicit mechanism early in adaptation [F(1,34) = 6.85, R^2^ = 0.168, p = 0.013]

We hypothesized that cognitive load influences the explicit, cognitive component of adaptation. Therefore, we examined the relationship between cognitive load and aiming strategy in early and late adaptation, as well as across the course of adaptation. There was a significant relationship between average cognitive load and aiming angle in Early Rotation [Fig. 4; F(1,34) = 6.85, R^2^ = 0.168, p = 0.013], where a higher cognitive load was related to a decreased use of an explicit, cognitive strategy early in adaptation. There were no significant relationships in Late Rotation [F(1,34) = 0.31, R^2^ = 0.009, p = 0.579] or throughout Rotation [F(1,34) = 1.97, R^2^ = 0.055, p = 0.170]. These results suggest that the increased cognitive load may affect the explicit, cognitive component of adaptation, specifically during the early stages of learning.

### Immediate and long-term retention and HMD-VR context transfer

**Fig. 5:**
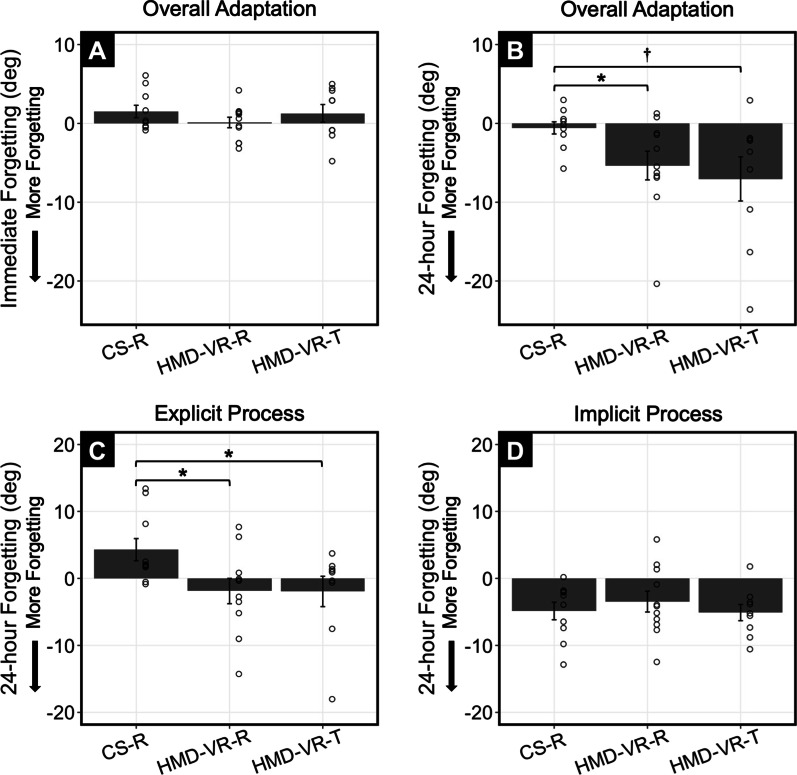
Results between HMD-VR and CS training environments on retention and context transfer. **A** Overall visuomotor adaptation at immediate forgetting was not significantly different between groups. **B** At delayed 24-h forgetting, there was more forgetting in HMD-VR-R than in CS-R [t(13.4) = 2.42, p = 0.031] and a trend of more forgetting in HMD-VR-T than in CS-R [t(9.2) = 2.23, p = 0.052]. **C** Differences in delayed 24-h retention could be explained by more explicit process forgetting in HMD-VR-R than in CS-R [t(18.9) = 2.47, p = 0.023] and in HMD-VR-T than in CS-R [t(15.0) = 2.23, p = 0.042]. **D** Forgetting of implicit process at delayed 24-h retention was not significantly different between groups. Dots represent individual participants and error bars indicate standard error. p < 0.05^*^, p < 0.1^†^

To examine retention and HMD-VR context transfer, we compared both immediate and delayed 24-h forgetting between CS-R, HMD-VR-R, and HMD-VR-T groups. There was no significant difference between groups for immediate forgetting [Fig. 5A; F(2,27) = 0.81, p = 0.455]. There was a trending difference between groups for delayed 24-h forgetting [Fig. 5B; F(2,27) = 3.01, p = 0.066]. Given our a priori hypothesis that cognitive load would affect long-term retention and context transfer, we further explored these results with post hoc analyses at delayed 24-h forgetting. HMD-VR-R showed significantly more forgetting than CS-R [t(13.4) = 2.42, p = 0.031], and HMD-VR-T showed a trend of more forgetting than CS-R [t(9.2) = 2.23, p = 0.052]. No differences were observed between HMD-VR-R and HMD-VR-T [t(14.2) = 0.51, p = 0.618]. While these results are not strong, they suggest that training in HMD-VR could possibly result in less long-term retention of the adaptation, independent of whether participants were measured in the same (HMD-VR-R) or in a different (HMD-VR-T) context.

We then examined whether the differences in long-term retention and context transfer could be explained by differences in explicit or implicit processes. There was a significant difference between groups for the amount of explicit processes forgotten after the 24-h retention interval [Fig. 5C; F(2,27) = 3.45, p = 0.046]. Post hoc analysis showed both HMD-VR-R and HMD-VR-T groups had significantly greater forgetting of the explicit process compared to CS-R [HMD-VR-R: t(18.9) = 2.47, p = 0.023; HMD-VR-T: t(15.0) = 2.23, p = 0.042]. No differences were observed between HMD-VR-R and HMD-VR-T [t(16.6) = 0.02, p = 0.984]. Separately, there was no significant difference between groups for the amount of implicit process forgotten after the 24-h retention interval [Fig. 5D; F(2,27) = 0.42, p = 0.663].

**Fig. 6:**
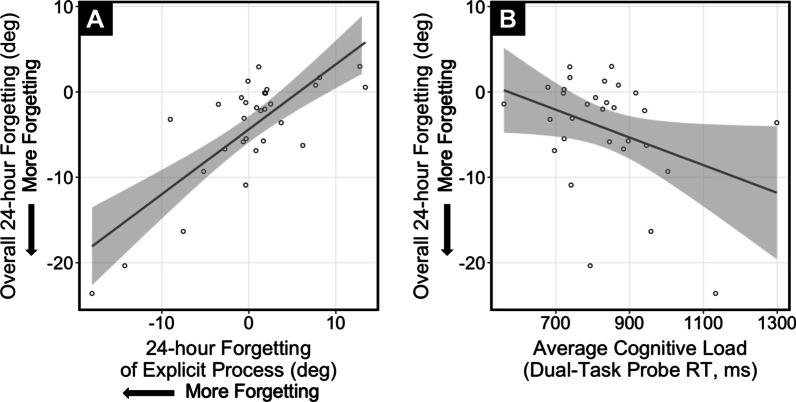
**A** Greater forgetting of the explicit process at delayed 24-h forgetting was related to decreased long-term retention, measured by more forgetting of the overall adaptation [F(1,28) = 43.38, R^2^ = 0.608, p < 0.0001]. **B** Increased cognitive load was related to decreased long-term retention, measured by more forgetting of the overall adaptation [F(1,28) = 4.31, R^2^ = 0.133, p = 0.047]

We then examined the relationship between forgetting of the explicit process and overall 24-h forgetting and found that greater forgetting of explicit processes was related to decreased long-term retention [Fig. 6A; F(1,28) = 43.38, R^2^ = 0.608, p < 0.0001]. These results suggest that the differences observed in the long-term retention between CS and HMD-VR groups can a explained by greater forgetting of explicit processes.

### Relationship between cognitive load and long-term motor memory formation

Since we hypothesized that cognitive load would influence long-term motor memories, we examined the relationship between cognitive load and 24-h forgetting across all groups. There was a significant relationship between average cognitive load and overall 24-h forgetting [Fig. 6B; F(1,28) = 4.31, R^2^ = 0.133, p = 0.047], where a higher cognitive load was related to increased 24-h forgetting. These results suggest that increased cognitive load may affect the retention of the learned adaptation.

## Discussion

The purpose of this study was to examine whether cognitive load increases in HMD-VR during visuomotor adaptation compared to a conventional computer screen (CS) environment, and whether increased cognitive load relates to long-term retention and context transfer. This was the first study to our knowledge that compared cognitive load in HMD-VR with known motor learning mechanisms and examined the relationship between cognitive load in HMD-VR with long-term motor memory formation. We found four main results. First, we showed that cognitive load is greater in HMD-VR compared to CS across adaptation. Second, we showed that higher cognitive load is related to decreased explicit, cognitive mechanisms, specifically early in adaptation. Third, we showed that visuomotor adaptation in HMD-VR leads to decreased long-term retention and context transfer, which appears to be due to greater forgetting of explicit processes. Fourth, we showed that increased cognitive load is related to decreased long-term motor memory formation. These findings have important implications for the development of clinical and motor learning applications in HMD-VR.

### Cognitive load during visuomotor adaptation is greater in HMD-VR than CS and related to decreased explicit processes early in adaptation

HMD-VR has been shown to increase cognitive load while performing complex motor skill tasks [16]. Here, we show that HMD-VR also increases cognitive load during a specific type of motor learning (i.e., visuomotor adaptation). Visuomotor adaptation is thought to be driven by explicit and implicit mechanisms. Explicit mechanisms are important early in adaptation and are thought to rely more on cognitive brain areas such as the dorsolateral prefrontal and premotor cortices [34–36]. Implicit mechanisms on the other hand develop over the course of adaptation and are the reflection of new visuomotor mappings driven by the anterior-medial cerebellum [34, 37]. These mechanisms are thought to work together in order to drive overall adaptation.

In this study, we found that early in adaptation, greater cognitive load was related to decreased explicit processes and that explicit processes—and subsequently, overall adaptation performance—were lower in HMD-VR than in CS. One interpretation of these findings is that greater cognitive load limits the use of explicit processes at the time when they are the primary drivers of overall adaptation. Put another way, increased cognitive load in HMD-VR limits the engagement of explicit processes specifically when they were most important for adaptation (i.e., early in adaptation). If this interpretation is true, then cognitive load may have the strongest affects early in the motor learning process. Motor learning in the real world has been shown to facilitate subsequent motor learning processes in HMD-VR, suggesting that HMD-VR may be more effectively used in later stages of motor learning [38]. Thus, initial training done without the use of HMD-VR may then increase the effectiveness of HMD-VR applications.

Another interpretation of these findings is that the engagement of explicit processes is limited at times when cognitive load is beyond working memory limits. We found that, while cognitive load was greater in HMD-VR than in CS across adaptation, overall cognitive load decreased over the course of training. Therefore, cognitive load may have limited the cognitive resources dedicated to the visuomotor adaptation task when it was most needed, early in adaptation. If this interpretation is true, then cognitive load may affect the motor learning process whenever the load on working memory is beyond working memory limits. HMD-VR applications may need to be designed to decrease cognitive load throughout training, or training in HMD-VR may need to be extended to reduce the early effects of cognitive load. Future work should systematically test these two hypotheses as both interpretations would affect how HMD-VR is effectively designed and used for motor learning applications.

Furthermore, recent evidence found suggests that a history of task errors, as opposed to a history of sensory prediction errors, is necessary for encoding memories [39]. In sensorimotor adaptation, explicit processes are thought to be driven by task errors and implicit processes are thought to be driven by sensory prediction errors [40]. Therefore, a decreased use of explicit processes early in adaptation in HMD-VR suggests that task errors were less relied on to update overall adaptation during this time. This could potentially explain why greater cognitive load related to decreased explicit processes could then result in decreased long-term retention.

Here we found that early in adaptation, the CS group had greater explicit learning and better overall adaptation compared to the HMD-VR group. These results are different from our findings in Anglin et al., 2017, where we found that compared to HMD-VR, CS had less explicit learning and similar overall adaptation over the course of adaptation, including early in adaptation. Explanations for these differences could be because of inter-subject variability, or because of differences in experimental design with the addition of the dual-task probe. One point in favor of the differences being due to different experimental designs is the differences in the response time in each experiment. That is, because of the dual-task probe, participants needed to wait after the initial presentation of the target before making their reach, increasing their response time to reach for the target. In a recent study by Langsdorf et al., 2021, it was found that forcing a wait period increased explicit learning compared to free reaching [41]. This finding is consistent with what can be found when comparing the CS groups between the present study and the study in Anglin et al., 2017. It is unclear why the HMD-VR group would not also experience an increase in explicit learning proportionately; however, this may be due to a ceiling effect given that the explicit processes are already increased due the HMD-VR environment.

Similar to our findings in Anglin et al., 2017, here we again found that at the end of adaptation, performance was the same whether training in HMD-VR or in CS, but the mechanisms driving performance were different between environments. Specifically, at the end of adaptation, the HMD-VR group showed a greater reliance on explicit mechanisms while the CS group showed a greater reliance on implicit mechanisms, although the net performance was the same across groups. Our interpretation of these results is that adaptation in HMD-VR relies more on explicit, cognitive strategies. If HMD-VR does rely more on explicit processes than implicit processes, then this can potentially explain why the performance was lower in HMD-VR than in CS early in adaptation, when explicit processes might have been affected by increased cognitive load.

One potential explanation for why cognitive load may be higher in HMD-VR could be how the brain processes vision for action. Vision for action is typically processed through the dorsal mode of control; however, artificial presentations of depth information in HMD-VR may cause a shift from a dorsal to ventral mode of control [42, 43]. A ventral mode of control is thought to be dependent on visual perception and increased cognitive processes and therefore could potentially explain increased cognitive load in HMD-VR during motor learning [44]. Separately, depth information has been found to uniquely affect explicit processes and therefore could also explain why HMD-VR may rely more on explicit, cognitive strategies [45]. Further research is needed to examine whether HMD-VR relies more on a ventral mode of control and whether this shift in control could explain a greater reliance of explicit processes in HMD-VR.

### Visuomotor adaptation in HMD-VR leads to decreased long-term retention and context transfer

In this study, we found that training in HMD-VR resulted in decreased long-term retention. Importantly, a decrease in retention occurred whether participants remained in an HMD-VR environment or transferred to a new context. Although the context transfer results reported here are relatively weak, this was likely due to large variability observed at delayed 24-h forgetting (Fig. 5B). Both findings from long-term retention and context transfer suggests that training in HMD-VR may lead to less efficient motor memory formation. That is, retention following training in HMD-VR cannot be explained by a context interference effect (i.e., better retention in the same environment as training), but is rather best explained by the training process itself.

Converging evidence suggests that explicit and implicit processes are homologous with the fast and slow processes of a dual-state model of sensorimotor learning [46]. The fast process generally dominates early in adaptation, responding strongly to error but exhibiting fast forgetting, while the slow process increases gradually, becoming stable over time and contributing to motor memory formation [47, 48]. Importantly, the slow process is thought to predict long-term retention, suggesting that implicit adaptation may also predict long-term retention. Implicit adaptation at the end of training was lower in HMD-VR than in CS and could potentially explain the decreased long-term retention and context transfer. We also found that long-term retention was related to greater forgetting of explicit processes. Taken together, these findings suggest that an increased reliance of explicit processes in visuomotor adaptation may lead to less efficient motor memory formation, explained by fast forgetting of explicit processes.

Given that in the present study a dual-task probe was combined with a typical visuomotor adaptation task in order to examine cognitive load, an important question is whether this modification might have altered the motor adaptation process. While this is a possibility, we would expect to see similar results in long-term retention and context transfer independent of the inclusion of the dual-task probe. This expectation is based on previous findings that the magnitude of implicit learning at the end of adaptation can be used as a predictor of retention (e.g., Joiner & Smith, 2008; Schweighofer et al. 2011). Both in the present study and in Anglin et al., 2017 which did not use a dual-task probe, the implicit learning at the end of adaptation was lower in HMD-VR than in CS. Therefore, because of this similarity we believe that the long-term retention and context transfer results in the present study were not meaningfully altered because of the dual-task probe modification.

### Cognitive load is related to decreased long-term motor memory formation

Cognitive load was found to be related to decreased motor memory formation. While this relationship was relatively weak, this finding indicates that the cognitive load increased by HMD-VR could directly affect the retention of a motor memory. Given that cognitive load during motor learning seems to have a negative effect on motor memory formation, HMD-VR motor learning applications should consider measuring cognitive load. Importantly, here we found that for both training environments, the cognitive load decreased over the course of adaptation. This finding has important implications for HMD-VR applications because it suggests that even though cognitive load may initially be high, it can decrease with practice or exposure. Motor learning applications and rehabilitation interventions in HMD-VR may need more training compared to conventional training environments to develop similar levels of motor skills. Future studies should look to see if continued training in HMD-VR could increase retention by decreasing cognitive load if people are given more time to practice.

### Limitations

A limitation of this study was the use of a computer screen to measure context transfer from HMD-VR. Using a computer screen allowed for a well-controlled study design as the only difference between HMD-VR and CS environments was the head-mounted display, which allowed us to control for any transfer effects that may have occurred due to a task change. However, future studies should examine whether increased cognitive load in HMD-VR during motor learning affects transfer to more dynamic, interactive real-world tasks such as manipulating physical objects, like a cup or a ball. Additionally, while visuomotor adaption is a specific type of motor learning, over-generalization to the domain of motor skill learning may not always be applicable [21]. Future studies should look to see if an increased cognitive load in HMD-VR during motor skill learning also affects long-term retention. Similarly, while the use of verbal reporting is a common way to measure explicit learning, this method has been shown to result in more explicit learning than other methods (e.g., exclusion) [49]. Future studies should examine whether the use of the exclusion method would reduce explicit learning in visuomotor adaptation in HMD-VR.

## Conclusions

We show that cognitive load was greater across visuomotor adaptation in HMD-VR compared to CS and related to decreased explicit processes early in adaptation. Cognitive load was also found to be related to decreased long-term motor memory formation, and visuomotor adaptation in HMD-VR resulted in decreased long-term retention and context transfer. These findings suggest that increased cognitive load in HMD-VR during motor learning may affect long-term motor memory formation. This study bridges motor learning mechanisms with a theoretical framework of cognitive load and examines the impact of cognitive load on motor memory formation. While these findings suggest that cognitive load may be increased in HMD-VR during motor learning, the reasons driving this increase is unclear. Future studies should aim to determine factors that may lead to increased cognitive load in HMD-VR motor learning.

## Supplementary Information

**Additional file 1.** Statistical analysis and Cognitive load is greater acrossvisuomotor adaptation in HMD-VR compared to CS.

## Data Availability

The data used in the current study are available from the corresponding author on reasonable request.

## Abbreviations

HMD-VR: Virtual reality using a head-mounted display
CS: Conventional computer screen
GLMM: Generalized Linear Mixed-Effect Model
RT: Reaction time
MT: Movement time

## References

[1] Levin MF (2020). What is the potential of virtual reality for post-stroke sensorimotor rehabilitation?. Expert Rev Neurother.

[2] Zimmerli L, Jacky M, Lünenburger L, Riener R, Bolliger M (2013). Increasing patient engagement during virtual reality-based motor rehabilitation. Arch Phys Med Rehabil.

[3] Levin MF, Demers M. Motor learning in neurological rehabilitation. Disabil Rehabil. 2020;1–9. 10.1080/09638288.2020.1752317 32320305

[4] Laver KE, Lange B, George S, Deutsch JE, Saposnik G, Crotty M. Virtual reality for stroke rehabilitation. Cochrane Database Syst Rev. 2017;2017. 10.1002/14651858.CD008349.pub4 PMC6485957 29156493

[5] Nemani A, Ahn W, Cooper C, Schwaitzberg S, De S (2018). Convergent validation and transfer of learning studies of a virtual reality-based pattern cutting simulator. Surg Endosc.

[6] Devos H, Akinwuntan AE, Nieuwboer A, Tant M, Truijen S, De Wit L (2009). Comparison of the effect of two driving retraining programs on on-road performance after stroke. Neurorehabil Neural Repair.

[7] Howard MC (2017). A meta-analysis and systematic literature review of virtual reality rehabilitation programs. Comput Human Behav.

[8] Müssgens DM, Ullén F (2015). Transfer in motor sequence learning: effects of practice schedule and sequence context. Front Hum Neurosci.

[9] Levac DE, Jovanovic BB. Is children’s motor learning of a postural reaching task enhanced by practice in a virtual environment? 2017 Int Conf Virtual Rehabil. IEEE; 2017. p. 1–7.

[10] Massetti T, Fávero FM, de Menezes LDC, Alvarez MPB, Crocetta TB, Guarnieri R (2018). Achievement of virtual and real objects using a short-term motor learning protocol in people with Duchenne muscular dystrophy: a crossover randomized controlled trial. Games Health J.

[11] Levac DE, Huber ME, Sternad D (2019). Learning and transfer of complex motor skills in virtual reality: a perspective review. J Neuroeng Rehabil.

[12] Juliano JM, Liew SL (2020). Transfer of motor skill between virtual reality viewed using a head-mounted display and conventional screen environments. J Neuroeng Rehabil.

[13] Levin MF, Magdalon EC, Michaelsen SM, Quevedo AAF (2015). Quality of grasping and the role of haptics in a 3-D immersive virtual reality environment in individuals with stroke. IEEE Trans Neural Syst Rehabil Eng.

[14] Magdalon EC, Michaelsen SM, Quevedo AA, Levin MF (2011). Comparison of grasping movements made by healthy subjects in a 3-dimensional immersive virtual versus physical environment. Acta Psychol (Amst).

[15] Anglin JM, Sugiyama T, Liew SL (2017). Visuomotor adaptation in head-mounted virtual reality versus conventional training. Sci Rep.

[16] Frederiksen JG, Sørensen SMD, Konge L, Svendsen MBS, Nobel-Jørgensen M, Bjerrum F (2020). Cognitive load and performance in immersive virtual reality versus conventional virtual reality simulation training of laparoscopic surgery: a randomized trial. Surg Endosc.

[17] Baumeister J, Ssin SY, Elsayed NAM, Dorrian J, Webb DP, Walsh JA (2017). Cognitive cost of using augmented reality displays. IEEE Trans Vis Comput Graph IEEE.

[18] Funk M, Kosch T, Schmidt A. Interactive worker assistance: Comparing the effects of in-situ projection, head-mounted displays, tablet, and paper instructions. UbiComp 2016—Proc 2016 ACM Int Jt Conf Pervasive Ubiquitous Comput. 2016;934–9.

[19] Orru G, Longo L, Longo L, Leva MC (2019). The evolution of cognitive load theory and the measurement of its intrinsic, extraneous and germane loads: a review. Commun Comput Inf Sci.

[20] Tseng YW, Diedrichsen J, Krakauer JW, Shadmehr R, Bastian AJ (2007). Sensory prediction errors drive cerebellum-dependent adaptation of reaching. J Neurophysiol.

[21] Krakauer JW, Hadjiosif AM, Xu J, Wong AL, Haith AM (2019). Motor learning. Compr Physiol.

[22] Miall RC, Wolpert DM (1996). Forward models for physiological motor control. Neural Networks Pergamon.

[23] Taylor JA, Ivry RB. Implicit and explicit processes in motor learning. Action Sci. 2013;63–87.

[24] Redding GM, Rader SD, Lucas DR (1992). Cognitive load and prism adaptation. J Mot Behav.

[25] Taylor JA, Krakauer JW, Ivry RB (2014). Explicit and implicit contributions to learning in a sensorimotor adaptation task. J Neurosci.

[26] Goh HT, Gordon J, Sullivan KJ, Winstein CJ (2014). Evaluation of attentional demands during motor learning: validity of a dual-task probe paradigm. J Mot Behav.

[27] Bouchard JM, Cressman EK (2021). Intermanual transfer and retention of visuomotor adaptation to a large visuomotor distortion are driven by explicit processes. PLoS ONE.

[28] Werner S, Strüder HK, Donchin O. Intermanual transfer of visuomotor adaptation is related to awareness. PLoS One. 2019;14. 10.1371/journal.pone.0220748 PMC6730885 31490953

[29] Vaswani PA, Shmuelof L, Haith AM, Delnicki RJ, Huang VS, Mazzoni P (2015). Persistent residual errors in motor adaptation tasks: reversion to baseline and exploratory escape. J Neurosci.

[30] Butcher PA, Taylor JA (2018). Decomposition of a sensory prediction error signal for visuomotor adaptation. J Exp Psychol Hum Percept Perform.

[31] McDougle SD, Taylor JA. Dissociable cognitive strategies for sensorimotor learning. Nat Commun. 2019;10. 10.1038/s41467-018-07941-0 PMC6318272 30604759

[32] Lo S, Andrews S (2015). To transform or not to transform: using generalized linear mixed models to analyse reaction time data. Front Psychol.

[33] French MA, Morton SM, Reisman DS (2021). Use of explicit processes during a visually guided locomotor learning task predicts 24-h retention after stroke. J Neurophysiol.

[34] Kim S, Ogawa K, Lv J, Schweighofer N, Imamizu H (2015). Neural substrates related to motor memory with multiple timescales in sensorimotor adaptation. PLoS Biol.

[35] Seidler RD, Noll DC (2008). Neuroanatomical correlates of motor acquisition and motor transfer. J Neurophysiol.

[36] Anguera JA, Reuter-Lorenz PA, Willingham DT, Seidler RD (2008). Contributions of spatial working memory to visuomotor adaptation. J Cogn Neurosci.

[37] Liew SL, Thompson T, Ramirez J, Butcher PA, Taylor JA, Celnik PA (2018). Variable neural contributions to explicit and implicit learning during visuomotor adaptation. Front Neurosci.

[38] Takeo Y, Hara M, Shirakawa Y, Ikeda T, Sugata H (2021). Sequential motor learning transfers from real to virtual environment. J Neuroeng Rehabil BioMed Central.

[39] Leow LA, Marinovic W, de Rugy A, Carroll TJ (2020). Task errors drive memories that improve sensorimotor adaptation. J Neurosci.

[40] Huberdeau DM, Krakauer JW, Haith AM (2015). Dual-process decomposition in human sensorimotor adaptation. Curr Opin Neurobiol.

[41] Langsdorf L, Maresch J, Hegele M, McDougle SD, Schween R (2021). Prolonged response time helps eliminate residual errors in visuomotor adaptation. Psychon Bull Rev Springer.

[42] Harris DJ, Buckingham G, Wilson MR, Vine SJ (2019). Virtually the same? How impaired sensory information in virtual reality may disrupt vision for action. Exp Brain Res.

[43] Ganel T, Goodale MA (2003). Visual control of action but not perception requires analytical processing of object shape. Nature.

[44] Goodale MA, Króliczak G, Westwood DA (2005). Dual routes to action: contributions of the dorsal and ventral streams to adaptive behavior. Prog Brain Res.

[45] Campagnoli C, Domini F, Taylor JA (2021). Taking aim at the perceptual side of motor learning: exploring how explicit and implicit learning encode perceptual error information through depth vision. J Neurophysiol.

[46] McDougle SD, Bond KM, Taylor JA (2015). Explicit and implicit processes constitute the fast and slow processes of sensorimotor learning. J Neurosci.

[47] Joiner WM, Smith MA (2008). Long-term retention explained by a model of short-term learning in the adaptive control of reaching. J Neurophysiol.

[48] Smith MA, Ghazizadeh A, Shadmehr R (2006). Interacting adaptive processes with different timescales underlie short-term motor learning. PLoS Biol.

[49] Maresch J, Werner S, Donchin O (2021). Methods matter: your measures of explicit and implicit processes in visuomotor adaptation affect your results. Eur J Neurosci.

