# Supplementary material for: Increased cognitive load in immersive virtual reality during visuomotor adaptation is associated with decreased long-term retention and context transfer
Source: J Neuroeng Rehabil. 2022 Oct 5;19:106. doi: 10.1186/s12984-022-01084-6 (PMC9532821; doi:10.1186/s12984-022-01084-6)
Supplement: Supplementary file 1 — Additional file 1. Statistical analysis and Cognitive load is greater acrossvisuomotor adaptation in HMD-VR compared to CS. [file 12984_2022_1084_MOESM1_ESM.docx]

**Supplementary Table 1. Summary of statistics for visuomotor adaptation training.**

| **Cognitive Load** | **t-value** | **df** | **p-value** | **CS** | **HMD-VR** |
| --- | --- | --- | --- | --- | --- |
| **Baseline** | -3.12 | 27.5 | 0.004 | 752±108ms | 883±138ms |
| **Rotation** | -2.96 | 30.4 | 0.006 | 744±107ms | 876±157ms |
| **Hand Angle** | **t-value** | **df** | **p-value** | **CS** | **HMD-VR** |
| **End of Baseline** | 1.55 | 34.0 | 0.130 | -0.53±1.18° | -1.45±2.38° |
| **Rotation** | 2.15 | 32.8 | 0.039^*^ | 44.96±3.24° | 41.82±5.45° |
| **Early Rotation** | 2.31 | 27.8 | 0.028^*^ | 30.87±11.5° | 20.46±14.9° |
| **Late Rotation** | -1.19 | 29.6 | 0.244 | 45.21±2.5° | 46.40±3.5° |
| **Aiming Angle** | **t-value** | **df** | **p-value** | **CS** | **HMD-VR** |
| **End of Baseline** | 0.48 | 18.8 | 0.640 | 0.09±1.2° | -0.09±1.0° |
| **Rotation** | -0.27 | 18.1 | 0.787 | 37.14±7.1° | 37.78±5.6° |
| **Early Rotation** | 2.09 | 23.7 | 0.047^*^ | 30.47±12.8° | 20.74±13.8° |
| **Late Rotation** | -2.28 | 14.5 | 0.038^*^ | 34.13±8.0° | 39.77±4.5° |
| **Implicit Adaptation** | **t-value** | **df** | **p-value** | **CS** | **HMD-VR** |
| **End of Baseline** | 1.11 | 30.0 | 0.277 | -0.62±1.62° | -1.36±2.3° |
| **Rotation** | 2.57 | 17.1 | 0.020^*^ | 7.82±4.5° | 4.04±3.3° |
| **Early Rotation** | 0.47 | 18.7 | 0.642 | 0.40±4.3° | -0.29±3.6° |
| **Late Rotation** | 2.14 | 17.0 | 0.047^*^ | 11.08±6.4° | 6.64±4.7° |

Mean and standard deviations are reported. p < 0.05^*^

**Supplementary Table 2. Summary of statistics for visuomotor adaptation retention.**

| **Hand Angle** | **F-value** | **df** | **p-value** | **CS-R** | **HMD-VR-R** | **HMD-VR-T** |
| --- | --- | --- | --- | --- | --- | --- |
| **Immediate Forgetting** | 0.81 | 2,27 | 0.455 | 1.51±2.5° | 0.11±2.2° | 1.27±3.4° |
| **24-hour Forgetting** | 3.01 | 2,27 | 0.066^†^ | -0.57±2.4° | -5.35±6.0° | -7.05±8.4° |
| **Explicit Process** | **F-value** | **df** | **p-value** | **CS-R** | **HMD-VR-R** | **HMD-VR** |
| **24-hour Forgetting** | 3.45 | 2,27 | 0.046^*^ | 4.30±5.2° | -1.89±6.3° | -1.95±6.8° |
| **Implicit Process** | **F-value** | **df** | **p-value** | **CS-R** | **HMD-VR-R** | **HMD-VR** |
| **24-hour Forgetting** | 0.42 | 2,27 | 0.663 | -4.87°±4.1° | -3.46°±5.2° | -5.10°±3.6° |

Mean $\boldsymbol{\pm}$ standard deviations are reported. p < 0.05^*^, p < 0.1^†^

**Supplementary Table 3. Results from GLMM examining cognitive load across the visuomotor adaptation task.**

| **Parameter** | **Estimate** | **Std. Error** | **t value** | **Pr (> \|z\|)** |
| --- | --- | --- | --- | --- |
| ${\hat{\boldsymbol{\beta}}}_{\boldsymbol{0}}$ Intercept | ﻿805.07 | 31.05 | 25.93 | < 0.0001^***^ |
| ${\hat{\boldsymbol{\beta}}}_{\boldsymbol{1}}$Cycle | -0.83 | 0.32 | -2.61 | 0.009^**^ |
| ${\hat{\boldsymbol{\beta}}}_{\boldsymbol{2}}$ Environment:HMD-VR | 167.39 | 38.41 | 4.36 | < 0.0001^***^ |
| ${\hat{\boldsymbol{\beta}}}_{\boldsymbol{3}}$ Cycle Number x Environment:HMD-VR | -0.80 | 0.42 | -1.89 | 0.059 |

Significance for fixed effects p < 0.0001^***^, p < 0.01^**^
